# The global environmental agenda urgently needs a semantic web of knowledge

**DOI:** 10.1186/s13750-022-00258-y

**Published:** 2022-02-17

**Authors:** Stefano Balbi, Kenneth J. Bagstad, Ainhoa Magrach, Maria Jose Sanz, Naikoa Aguilar-Amuchastegui, Carlo Giupponi, Ferdinando Villa

**Affiliations:** 1 Basque Centre for Climate Change (BC3), Scientific Campus of the University of the Basque Country, Sede Building 1, 1st floor, Barrio Sarriena S/N, 48940 Leioa, Bizkaia Spain; 2 IKERBASQUE, Basque Foundation for Science, Plaza Euskadi, 5, 48009 Bilbao, Spain; 3 U.S. Geological Survey, Geosciences and Environmental Change Science Center, Denver, CO USA; 4 World Wildlife Fund, Washington, DC USA; 5 Department of Economics, Ca’ Foscari University of Venice, Venice, Italy

**Keywords:** Global challenges, Sustainability, Artificial intelligence, Semantics, Knowledge integration and synthesis

## Abstract

Progress in key social-ecological challenges of the global environmental agenda (e.g., climate change, biodiversity conservation, Sustainable Development Goals) is hampered by a lack of integration and synthesis of existing scientific evidence. Facing a fast-increasing volume of data, information remains compartmentalized to pre-defined scales and fields, rarely building its way up to collective knowledge. Today's distributed *corpus* of human intelligence, including the scientific publication system, cannot be exploited with the efficiency needed to meet current evidence synthesis challenges; computer-based intelligence could assist this task. Artificial Intelligence (AI)-based approaches underlain by semantics and machine reasoning offer a constructive way forward, but depend on greater understanding of these technologies by the science and policy communities and coordination of their use. By labelling web-based scientific information to become readable by both humans and computers, machines can search, organize, reuse, combine and synthesize information quickly and in novel ways. Modern open science infrastructure—i.e., public data and model repositories—is a useful starting point, but without shared semantics and common standards for machine actionable data and models, our collective ability to build, grow, and share a collective knowledge base will remain limited. The application of semantic and machine reasoning technologies by a broad community of scientists and decision makers will favour open synthesis to contribute and reuse knowledge and apply it toward decision making.

## Complex global issues, fragmented knowledge

The global environmental agenda includes diverse internationally agreed-upon goals encompassing varied social and ecological challenges (e.g., climate change, biodiversity conservation, economic cooperation, migration and most recently, pandemic response). Almost every nation, non-governmental organization and large corporation participates in initiatives addressing one or more of these policy goals. However, our ability to deliver timely and accurate scientific evidence to address these problems remains limited.

The lack of tangible progress across these global challenges relates in part to their nature as “wicked problems”—intertwined, multistakeholder and with potential solutions dependent on subjective, competing interests. Further, tightly linked issues like climate change and biodiversity loss are dealt with in separate policy forums (e.g., Convention on Biological Diversity (CBD), Intergovernmental Panel on Climate Change (IPCC), Intergovernmental Science-Policy Platform on Biodiversity and Ecosystem Services (IPBES)) and strategies (e.g., European Union Biodiversity Strategy, European Union Adaptation Strategy). Addressing them efficiently requires unprecedented integration and synthesis of evidence (including data and models produced by the scientific community, but also traditional and stakeholder knowledge) that can lead to broadly shared solutions among a diverse range of stakeholders. Here, we discuss how the current lack of knowledge integration and evidence synthesis protocols and technologies is a critical constraint limiting the design and implementation of policies to support global sustainability efforts; other barriers including lack of adequate multiscale governance, power asymmetry, political will, the digital divide [1] and risks of unethical Artificial Intelligence (AI) use [2, 3] fall outside the scope of this article.

Currently, we are witnessing increased demand for open science through the promotion of the FAIR principles aimed at making data and models Findable, Accessible, Interoperable and Reusable [4]. However, despite advances in open data and scientific methods, tools enabling interoperability (i.e., the “I” in FAIR) remain time- and resource-intensive [5]. Further, open science has been incompletely adopted within the field of evidence synthesis [6, 7]. As volumes of data increase rapidly, information reuse remains compartmentalized within pre-defined scales and fields, too rarely building its way up to collective knowledge.

Additionally, notwithstanding the rapid growth of interdisciplinary science, scholars are still often incentivized to study particular scientific fields using disciplinary methods and worldviews. Despite substantial progress, barriers to interdisciplinarity remain in funding, publishing, and communication, which can limit collaboration and knowledge sharing [8]. In proposing the idea of an open synthesis community, Nakagawa et al. [7] note the impossibility of keeping up with the deluge of scientific information, thus methods are needed to automate the synthesis of research evidence, while simultaneously respecting its complexity.

Indeed, knowledge integration and evidence synthesis at the speed and depth required by the global environmental agenda lie beyond the capacity of today’s distributed human intelligence and can benefit from the assistance of computer-based intelligence. To this end, we argue that an AI-facilitated approach based on semantics and machine reasoning (see Box 1 for key definitions) offers a feasible path forward *to connect the data and digital technologies that are now held by the academic, public and private sectors, so they can generate real-time insight about the state of the planet at any scale*. However, such an approach will require coordination across the scientific community to create, implement and deliver truly FAIR scientific workflows for decision-making.

Box 1. Definition of key concepts in this article

**Table:** 

**Artificial Intelligence (AI):** the science and engineering underlying the development of machines, especially computer programs, capable of performing activities normally thought to require intelligence. AI encompasses approaches including machine reasoning, semantic annotation, machine learning, and others
**Interoperability:** the ability of data or tools from independent resources to integrate or work together with minimal effort [4]. Interoperability can be achieved with compatible data formats and communication protocols (syntactic interoperability) or data transfers where a receiving system can properly identify the meaning of exchanged data, reusing it appropriately (semantic interoperability [11])
**Knowledge integration:** the process of appropriately combining independently produced scientific data and models, by knowing when, where and how to appropriately re-use them
**Machine learning:** the use of various algorithms to uncover patterns (e.g., correlation or clustering) in large datasets. Without structured inputs to extract patterns, machine-learning systems cannot solve new problems that have no apparent relation to their prior knowledge. Machine learning is currently the most widely used form of AI
**Machine reasoning (i.e., machine-operated logical inference using formalized semantics):** applied to a semantically annotated knowledge base, machine reasoning can support automated validation and linking of data and models using logic to assemble them into useful structures for computation. Reasoning systems can tackle new problems and build higher-level knowledge using deductive and inductive reasoning
**Semantics:** the formalization of knowledge in terms of logical declarations and axioms, collected into *ontologies* (which define concepts and the relations between them), breaking knowledge into modular components. Semantic annotation can label scientific data and models with well-defined categories linked by clearly bounded logical relationships and can play a key role in knowledge integration

## A semantic web of knowledge

Our world is undergoing dramatic digital transformations with data generated at a never-before seen volume and velocity [1, 2]. These include data generated by mobile devices, satellite and ground sensors, social media and citizen-science platforms, coupled with cloud and high-performance computing and machine learning. Despite these technological, scientific and societal developments, we are not keeping pace with humanity’s greatest challenges to progress towards solutions.

Additionally, although our understanding of planetary-scale processes has improved, we are far from being able to accurately track key dynamics and critical thresholds across diverse scales and drivers. Key processes, entities (e.g., nations, watersheds, households, ecological communities) and their interdependencies across scales are far too complicated for individual human brains to disentangle [9]. Simultaneously, today’s repositories of human intelligence, such as the scientific publication system, fall short in connecting the pieces of knowledge produced by different fields. AI assistance offers a path forward.

To provide needed decision support, AI must ultimately simulate Earth as a real-time, dynamic system composed of nested social-ecological systems. A “digital twin Earth” has been included in recent communications on the European Green New Deal [2]. The idea of building a simulation of the planet has been proposed in different forms by global (e.g., UN Environment, Group on Earth Observations), European and U.S. institutions (e.g., European Commission and European Spatial Agency, the U.S. Geological Survey and NASA), and the private sector (e.g., Microsoft AI for Earth, Google Earth Engine). However, these are mostly understood as massive machine-learning efforts built on Earth observations from a wide range of sources, with limited attention paid to semantics and machine reasoning. Recently, a global digital ecosystem for the planet was proposed by the UN Environment Programme as “a complex distributed network” consisting of four key elements: (1) data, (2) algorithms and analytics (i.e., models), (3) supporting technological infrastructure and (4) insights and applications [10]. A primary technological bottleneck in building such cyberinfrastructures, which aim to bring data, models and processing power together in various clouds, is *how to make independently produced data and models seamlessly interoperable?*

We argue for a solution built upon semantics and machine reasoning [11, 12] (see Box 1). AI research points toward a convergence of technologies (machine reasoning and machine learning, geospatial intelligence, data analytics and visualization, sensors and smart connected objects) to sustain governance platforms in natural and social systems [13]. *Machine reasoning* is driven by facts and knowledge that can be used to validate and link information using logical inference [14]. Concepts, entities, their relationships and (to some extent) behaviours are described in shared documents (*ontologies)* that establish a logical foundation to consistently annotate web-accessible data and model resources. This knowledge base, paired with AI, could bring the FAIR principles to full fruition. Such AI can help harness the complexity of integrating independently produced data and models with the goal of maximizing human well-being and restoring ecosystem functioning [15]. Multidisciplinary *semantics* that are explicitly engineered to support reasoning can make human knowledge interoperable at a large scale and in distributed fashion, so that machines can assemble it to address complex social-ecological issues. Widespread use of semantics would vastly improve the status quo, where inconsistent and imprecise use of terms across different fields impedes the synthesis of scientific evidence (e.g., [16]).

By labelling peer-reviewed, web-based scientific information in ways readable by both humans and computers, and using common standards for machine-actionable data and models, machines can search, organize, reuse and combine information quickly and in novel ways—i.e., a semantic web of knowledge [17, 18]. Achieving this will require several actions on the part of scientists that go beyond the state of the practice for today’s open science. For example, the Artificial Intelligence for Environment and Sustainability Project (ARIES, [19]) described below provides infrastructure to enable these steps. Specifically, key elements in ARIES enable (1) data and model developers to expose and maintain knowledge resources as independently hosted and open web services using networked architecture, open standards and application programming interfaces (APIs); (2) consistent semantic annotation practices that can be applied by data and model developers, who can concurrently participate in the development of ontologies, while producing more modular models carrying documentation and appropriate reuse conditions; and (3) a vision of a peer-to-peer network hosting content available for machine-actionable synthesis, with institutions maintaining interoperable data and model resources over time. More details on each of these steps can be found in Villa et al. [20].

This approach connects existing, web-accessible data and models, so that new multidisciplinary scientific knowledge can be generated from them on demand, complementing much slower human-driven model coupling and reuse [5]. AI-supported, on-the-fly assembly of scientific workflows enables the incorporation of newly produced data sources as they become available on the network, reducing latency and providing a path toward much needed near-real-time modelling. Widely used semantics call for open, transparent and well-documented models, forcing a simple and modular model coding style where encapsulated documentation can be made mandatory. In this way, integrated computational workflows can collect and process information about each individually documented modelling component, delivering fully transparent assessments to model users [20].

In the face of widespread use of, and publicity for, “big data-driven” machine learning [9], we believe wider understanding and use of semantics and machine reasoning in scientific modelling is critical to addressing today’s sustainability challenges. Approaches such as ARIES have demonstrated how semantics can maximize data and model reusability and interoperability when assessing ecosystem services and, more generally, in modelling complex human-nature interactions and their consequences.

Notably, ARIES has been applied to the System of Environmental Economic Accounting (SEEA)—an international statistical standard used to measure linkages between national economic accounts and natural capital stocks and ecosystem service flows in physical and monetary terms, as well as information on the extent and condition of ecosystems [21]. ARIES for SEEA was released in April 2021, and it is accessible at https://seea.un.org/content/aries-for-seea. It provides a common platform to make data and models interoperable and improve the ability of National Statistical Offices to automate the compilation of environmental-economic accounts and related indicators, which requires the ability to integrate national statistics and spatial data and models. ARIES for SEEA thus demonstrates a path forward for better synthesizing the information required to monitor complex linked social-ecological systems through indicators such as the Sustainable Development Goals.

Semantic-driven integration technologies, such as ARIES, offer six critically needed advantages to twenty-first century interdisciplinary science and decision-making, and pioneer a new generation of distributed digital infrastructure to integrate independently produced data and models served online—a web of scientific observations with the capability to:

1. Combine independently produced scientific products into workflows that would be too complex for individual humans to conceive, validate and navigate.
2. Integrate different modelling paradigms from simple (e.g., deterministic and probabilistic models) to complex approaches (e.g., agent-based and networks) depending on context and scale.
3. Rescale smartly across scales, from local to global, promoting adaptive solutions that are automatically customized to the scale of observation.
4. Flexibly incorporate the best-available knowledge, from curated global public datasets to “big data” to user-provided data.
5. Adopt common, non-ambiguous semantics in both the implementation and delivery of products.
6. Track quality and uncertainty throughout modelling workflows.

## Toward a global digital commons

**Fig. 1:**
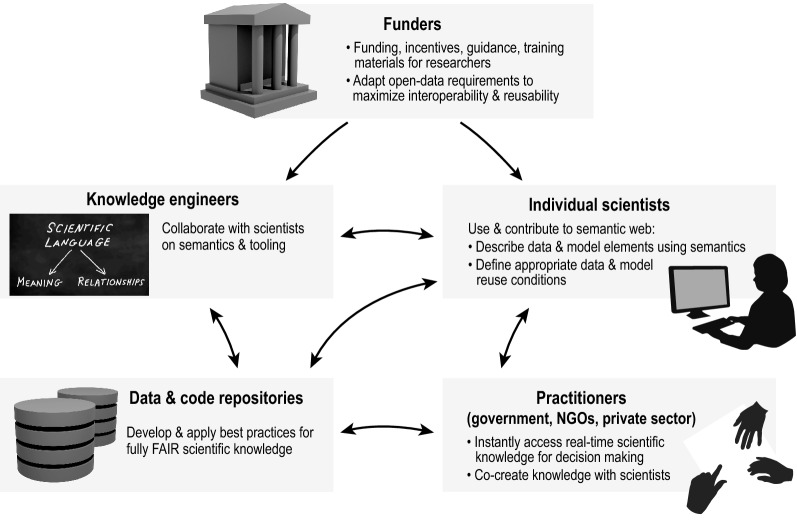
Roles in the transition to a semantic web of knowledge

Today’s open science infrastructure—public data and model repositories—provides an important starting point toward the vision of an integrated knowledge landscape outlined above. However, the lack of shared semantics hinders our collective ability to fully exploit and continuously expand the existing knowledge base. Coordination across the entire scientific community will be needed to achieve widespread use of a shared semantic system (Fig. 1). This will entail (1) incentives from funders for creation and use of semantically interoperable systems, driving (2) substantially closer collaboration between domain scientists, knowledge engineers and next-generation data and code repositories, which leads to (3) everyday, AI-assisted use of the growing knowledge base by both scientists and decision makers. With substantially lowered barriers for non-semantic experts to contribute knowledge, and AI bearing the largest share of the data and model interoperability burden, unprecedented access to connected scientific knowledge should be possible. The use of semantics in modelling is most powerful as an intentional, collaborative process that effectively integrates the knowledge of individual scientists and data providers. In other words, modelling processes and products, and the semantics to describe them, should be vetted by a large and multidisciplinary scientific community during their development. Through a semantics-driven approach, the scientific community can support the environmental agenda by contributing to a global *digital commons* of data and models in a Wikipedia-like fashion.

A working multi-scale Earth system platform, dedicated to evidence synthesis for monitoring the global social-ecological challenges, could be reached by generalizing observed patterns based on data collected by well-established networks (e.g., from Long Term Ecological Research stations). This can be initially achieved through a representative set of case studies that can serve as archetypes for machine learning and transferring the knowledge acquired with data-driven statistical methods. Relative to the *status quo* of manual model coupling and AI focused solely on machine learning, semantic knowledge integration offers a path to better address long-standing challenges related to the exploration of alternative futures, tipping points, and discontinuities. Further, such a global platform could incorporate multidimensional values, including heterogeneous stakeholders’ preferences, using interactive technologies (e.g., data viewers, graphical editors), which can account for subjective preferences when interpreting model outputs.

Nakagawa et al. [7] describe a vision for how improved interoperability can help fuel an “evidence revolution” in which old and new evidence can be quickly and transparently synthesized—a task to which a semantic web of knowledge is well suited. As a scientific community, our main challenge is to quickly adopt and provide an integrated and scalable solution to support decision-making for a more sustainable planet, while navigating fast-moving and interconnected global crises. Semantics and machine reasoning offer a proven way forward, but the benefits they offer for synthesis urgently require more widespread understanding and coordination of their use across the scientific and policy communities.

## Data Availability

Data sharing is not applicable to this article as no datasets were generated or analysed during the current study.

## Abbreviations

AI: Artificial Intelligence
API: Application programming interface
ARIES: Artificial Intelligence for Environment and Sustainability
CBD: Convention on Biological Diversity
EC: European Commission
FAIR: Findable, accessible, interoperable, reusable
IPCC: Intergovernmental panel on climate change
IPBES: Intergovernmental science-policy platform on biodiversity and ecosystem services
NASA: National aeronautics and space administration
SEEA: System of environmental economic accounting
UN: United Nations
US: United States
